# Evaluating process fidelity during the implementation of Group Antenatal Care in Mexico

**DOI:** 10.1186/s12913-020-05430-z

**Published:** 2020-06-18

**Authors:** Evelyn Fuentes-Rivera, Ileana Heredia-Pi, Zafiro Andrade-Romo, Jacqueline Alcalde-Rabanal, Lourdes Bravo, Laurie Jurkiewicz, Blair G. Darney

**Affiliations:** 1 Center for Health Systems Research, National Institute of Public Health, Av. Universidad 655, Col. Santa María Ahuacatitlán, 62100 Cuernavaca, Morelos Mexico; 2 Department of ObGyn & Reproductive Sciences, UCSF/SFGH, San Francisco, CA USA; 3 OHSU, Oregon Health & Science University, Portland, OR USA; 4 National Institute of Public Health, Cuernavaca, Mexico

**Keywords:** CenteringPregnancy, Model fidelity, Mexico, Process fidelity, Group antenatal care, G-ANC, Implementation

## Abstract

**Background:**

CenteringPregnancy (CP) is a group antenatal care (G-ANC) model that has proven beneficial for mothers and their newborns. We conducted a feasibility study beginning in 2016 as part of the Mexican effort to implement G-ANC locally. This study reports on fidelity to the essential elements of CP during its implementation in Mexico.

**Methods:**

We collected prospective data using a standardized checklist at four primary-care centers that implemented our adapted G-ANC model. We performed a descriptive analysis of fidelity to 28 processes per G-ANC session (71 sessions made up of 10 groups and 129 women across 4 health centers). We calculated fidelity to each process as a proportion with 95% confidence intervals. We present overall results and stratified by health center and by facilitation team.

**Results:**

Overall fidelity to the G-ANC intervention was 82%, with variability by health center (78–88%). The elements with the highest fidelity were having space for activities such as checking vital signs, conversation in a circle, and medical check-ups (100% each) and the element with the lowest fidelity was using music to enhance privacy (27.3%). Fidelity was not significantly different by center.

**Conclusions:**

Our study suggests good model fidelity during the implementation of G-ANC in Mexico. Our findings also contribute useful information about where to focus efforts in the future to maintain and improve G-ANC model fidelity.

## Background

**Fig. 1:**
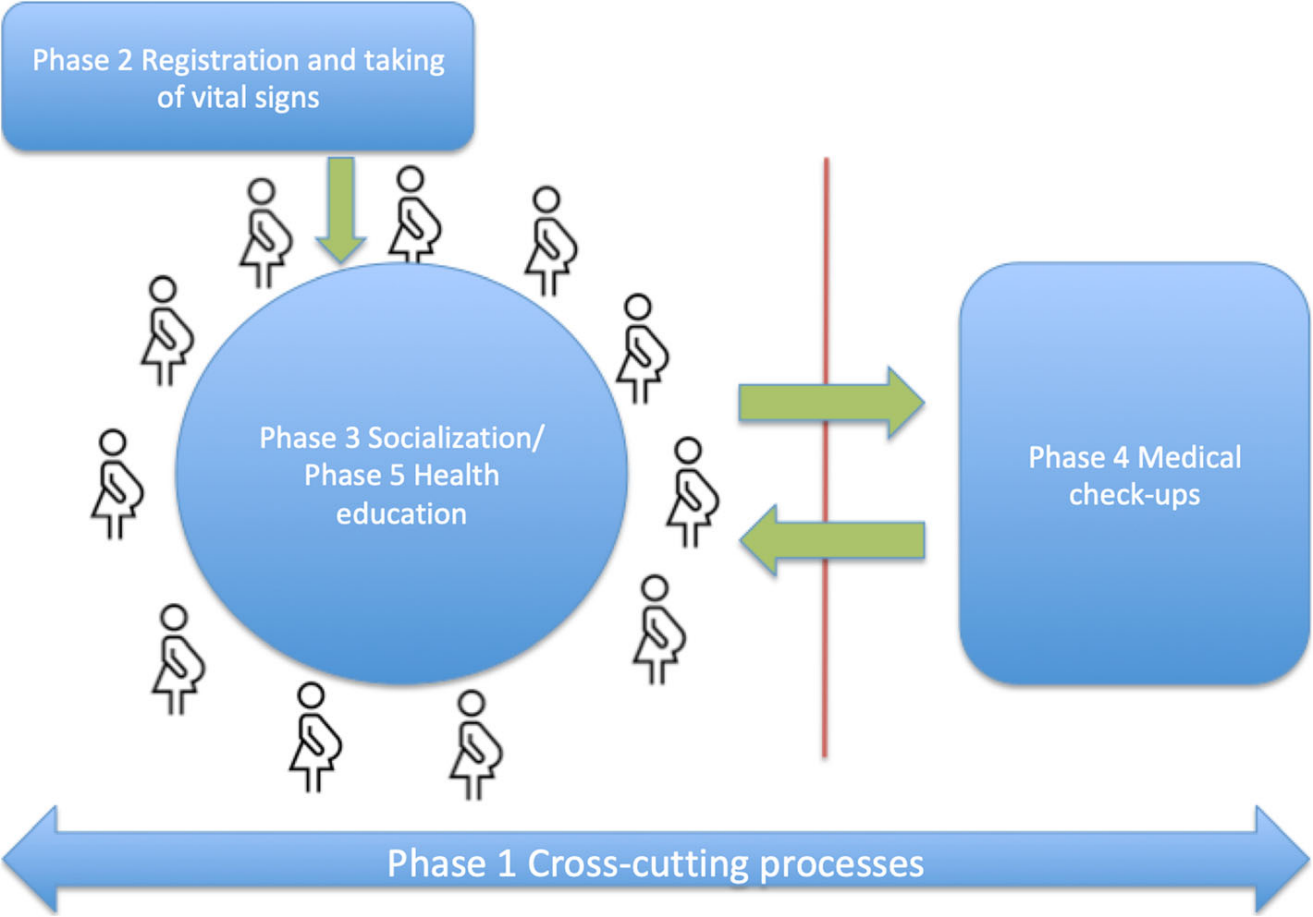
Care cycle. In each session, participants follow a care cycle. The first phase includes processes that are developed during the entire session – cross-cutting processes; the second phase includes registration and self-care processes; third phase refers to socializing or building of social networks; the fourth phase refers to medical check-ups; and the fifth phase includes health education

CenteringPregnancy (CP) is a group antenatal care (G-ANC) model that replaces traditional individual care [1]. This model is offered by diverse health care providers including physicians, nurses and midwives. Providers act as facilitators of groups of 8–12 women of similar gestational age and meet 8–10 times in sessions of approximately 2 hours during a women’s pregnancy. In each session, women follow a four-phase cycle, where several processes are completed (Fig. 1). This cycle includes: (a) registration and self-care: as women arrive, they sign in and check their own vital signs; (b) socializing or building of social networks: women sit in a circle and spend time chatting freely with their peers; (c) medical check-up: in parallel with the second phase, each woman exits the circle to have an individual consult with the doctor or midwife, who conducts individual physical examinations in the same space as the group; and (d) health education: once the round of check-ups has been completed, the women and facilitators form a circle and are offered information about pregnancy through a participatory and non-hierarchical approach [2] (Fig. 1).

CP and related G-ANC models (adaptations of CP to national standards and norms in each country) have shown maternal and neonatal benefits in more than 22 countries and with specific population groups. Recently, we published a narrative review of the literature in relation to its effectiveness evaluated in different contexts of implementation [3]. Evidence showed better outcomes in terms of the knowledge that pregnant women acquire during group prenatal care, nutrition, breastfeeding, changes during pregnancy, family planning; as well as increased use of family planning services in the postpartum period [3].

The essential elements of CP (Supplemental material Table S1) [4] revolve around format or structure as well as processes of care. For example, the group sessions should take place in a circle, and the individual medical check-ups also happen within the shared space, off to the side. Self-care is also a fundamental aspect of G-ANC; pregnant women become actively involved in activities such as self-monitoring blood pressure and weight. Another important component of group care relates to the educational environment, especially the role of facilitators. While sessions should follow a general plan, it should be flexible enough for women to address the topics they are more interested in, and facilitators should enable the space to allow interaction between women and allow the contributions of every group member. CP and other G-ANC models share the same essential elements, however G-ANC models may differ in some details according to their own process of adaptation in different contexts [5, 6].

During each CP session, the education discussion comprises the majority of group time. Education and support are gained from interactions with group facilitators, guest speakers, and pregnant women who are members of the group. The model philosophy is aimed at promoting health and facilitating women’s trust in their own abilities [7]. Pregnant group members gain confidence, feel more comfortable asking questions, learn that others are in similar situations, and develop camaraderie with fellow group members. Women learn as much or more from other women in the group, which contributes directly to increasing each woman’s sense of empowerment, particularly in socially disadvantaged women or women with low health literacy [1, 7].

In Mexico, as in many other countries, conventional individual antenatal care is often of low quality, including access, timeliness, equity, and continuity of care [8], whether measured by frequency of visits or by having the same health care provider [9]. At the national level, disparities persist in the continuity of maternal care (a composite of antenatal care initiation, frequency, content of care, institutional birth, and postpartum contraception), especially among women of lower socioeconomic status [10].

The CP model of care has the potential to improve the quality and continuity of antenatal care in Mexico. In 2016, we initiated a study to adapt the CP model to the Mexican context and measure the feasibility and acceptability of the model among users and health center staff during implementation. More information about the adaptation and implementation of the model is detailed elsewhere [6], but in brief, our process of adapting and implementing the CP model had 6 steps (Ministry of health buy-in and training of Mexican team, adaptation of content and format of the CP model, site selection process, initial training of health center staff, pilot and implementation) and occurred in four primary-care centers of two Mexican states (Morelos and Hidalgo) from 2016 to 2018 (Supplemental material Table S2).

When a new and complex intervention is implemented, measuring fidelity to the intervention is useful; observed impacts of the intervention may be moderated by degree of fidelity to the intervention as designed [11]. The evaluation of fidelity, or compliance with a model, indicates whether an intervention is being implemented as originally planned [11]; this offers useful guidance to those interested in its replication and facilitates studying the impact of complex interventions [11, 12]. Evaluation of intervention fidelity also identifies which components of an intervention achieve the highest degree of fidelity and which do not, which can be used to develop strategies to adjust or correct deviations from the intervention [12, 13]. The literature describes various dimensions of fidelity; in this study we focused on process fidelity, which measures whether the strategies and skills used to implement the intervention are those defined in the design [13, 14]. Basically, we focused on assessing adherence, understood as whether “a program service or intervention is being delivered as it was designed or written” [11, 15]. If evidence-based health interventions are to translate successfully into improved medical practices, ensuring fidelity is crucial [16]. In complex interventions, fidelity must focus on the process of the intervention more than on individual components [17]. Literature does not provide a standard procedure to measure fidelity in health interventions. Some studies measure fidelity via on-site observation or audio recordings [18, 19]. Other studies use surveys and qualitative interviews with the staff in charge of implementation at specific moments of the intervention [20, 21]. However, the literature consistently present fidelity as the compliance rates achieved by the recommended or designed themes and techniques utilized during intervention [14, 18–21].

Based on our experience of adapting and implementing the CP model in Mexico, the purpose of this study was to evaluate fidelity to our adapted G-ANC model during its implementation in four public primary-care health centers in two Mexican states.

## Methods

### Study design

We undertook a prospective descriptive study at four public-sector (Ministry of Health) primary-care centers that implemented the adapted G-ANC model. As the model was implemented in public-sector primary-care centers the target population was low income women who resided in the same geographical area as the clinic. Our unit of analysis was the G-ANC session, and we included all sessions between June 2016 and February 2018. Each primary-care center had at least one facilitation team (3 centers had two teams), the facilitation teams developed one or more groups of pregnant women, these groups functioned as a cohort and included at least 7 sessions. The 71 sessions took place within ten separate G-ANC groups that included 129 women. Table 1 describes the population. The sessions were led by seven facilitation teams, composed of a physician (the team leader), nursing staff and, in some cases, social workers or psychologists. Four of the seven teams were led by women and the nursing staff was predominantly female.

**Table 1:** Groups, facilitation teams and characteristics of participants by health care center

Implementation characteristics	Center 1	Center 2	Center 3	Center 4	Global
No. of groups	3	2	3	2	10
No. of participating women^a^	42	27	34	26	129
No. of women who attended ≥5 of the 7 or 8 possible sessions (% of total women recruited)	36 (85.7%)	19 (70.4%)	28 (82.3%)	24 (92.3%)	107 (82.9%)
No. of trained facilitation teams	2	1	2	2	7
Average age of expectant women	23.85	25.76	21.28	21.27	23.03
	(21.7–26.0)	(23.3–28.2)	(19.4–23.1)	(19.1–23.4)	(21.9–24.1)
Proportion of adolescent women (< 25 years)	65.85	36.00	75.00	76.92	64.52
	(50.7–81.0)	(15.8–56.2)	(59.1–90.9)	(59.6–94.3)	(56.0–73.0)
Mean of gestational age at the outset	18.19	17.67	17.66	15.3	17.34
	(16.2–20.2)	(15.6–19.7)	(15.9–19.4)	(13.2–17.4)	(16.4–18.3)
Parity (proportion of nulliparous women)	41.46	32.00	59.37	50.00	45.97
	(25.7–57.2)	(12.3–51.)	(41.4–77.4)	(29.4–70.6)	(37.1–54.9)

^a^Women who attended ≥1 session

### Data collection

Data were collected through a standardized checklist developed by team members with experience and expertise in the CP model. Every session two members of our research team observed the session and completed the standardized checklist at the end after giving some feedback to the facilitation team. The feedback was based in key elements on the checklist; research team members reviewed the elements with the facilitation team in order to facilitate reflection about their own accomplishment of the key elements during the session. Although the feedback was based in key elements, it was conducted as a conversation in which research team members try to evoke what the facilitation team learned in workshop and what they did in session. The observers were the same for all sessions of each group. They were trained in the same workshops as the facilitators and received a special training to complete the checklist. They were also in contact with our national and international experts to resolve any doubts about the model. Our 28-item fidelity checklist (ten binary and 18 Likert-scale) is based on the essential elements and on the fidelity checklist of the original CP model [22]. (Supplemental material Table S1).

### Analysis

We classified the 28 items into five groups, following the phases of a group care session (Fig. 1). The first group of seven items was made up of processes that were relevant or impacted the entire session – cross-cutting processes. We classified six items as part of phase two: taking and recording vital signs. Phase three, socialization, includes two items, while phase four, focused on the place used for the physical examination, includes three items. Phase 5, health education, corresponded to ten items on the instrument (Table 2 and Supplemental material Table S3).

**Table 2:** Process fidelity during the implementation of Group Antenatal Care in Mexico: results by phase and for the entire intervention according to trained facilitation teams

Phases	All sessions	Facilitation teams						
		Center 1		Center 2	Center 3		Center 4	
		Team 1	Team 2	Team 3	Team 4	Team 5	Team 6	Team 7
	*N* = 71	*N* = 15	*N* = 7	*N* = 14	*N* = 13	*N* = 7	*N* = 8	*N* = 7
Overall process fidelity	83.18	83.19	83.52	88.17	75.00	82.61	69.19	88.76
Phase 1 Cross-cutting processes	77.71	83.56	95.24	80.56	69.70	83.72	52.00	80.95
Phase 2 Registration and taking of vital signs	91.97	87.50	91.67	98.41	88.89	97.22	92.68	91.43
Phase 3 Socialization	94.37	90.00	92.86	96.43	92.31	100.00	93.75	100.00
Phase 4 Medical check-up	73.09	68.57	66.67	89.47	58.82	100.00	58.33	66.67
Phase 5 Health education	78.61	83.22	75.36	85.40	72.66	65.71	66.25	95.71

*N* Number of sessions

Our outcome was overall process fidelity, calculated as a proportion. As we looked for complete fidelity, we collapsed the Likert scale items to binary variables (1 when the response was “always” or “all,” depending on the item, and 0 if not). For example, the item *Has the facilitator guided but not controlled the conversation?* had the following response options: never, sometimes, normally, most of the time, and always. The transformed variable was assigned a value of 1 when the response was *Always,* and 0 when it was any of the other options. We obtained a proportion of accomplishment for every item, adding all sessions where the item/process had 1 (the positive extreme of the Likert scale) and dividing this addition by all applicable sessions for the item. To calculate fidelity by phase we calculate a mean of all proportions of accomplishment of the items included in each phase. To look for time variability within the groups, we also calculated fidelity by session for every group by adding all items/process that had 1 in each session and dividing this addition by the total of items for the session. We calculated 95% confidence intervals by phase and by site. We present overall results and stratified by site and by facilitation team.

The Comité de Ética en Investigación of the Instituto Nacional de Salud Pública de México has approved this project (#1756). All women enrolled in the G-ANC groups signed a written inform consent approved by the Comité de Ética en Investigación of the Instituto Nacional de Salud Pública de México on May 24th, 2016 with number of approval N-69.

## Results

**Fig. 2:**
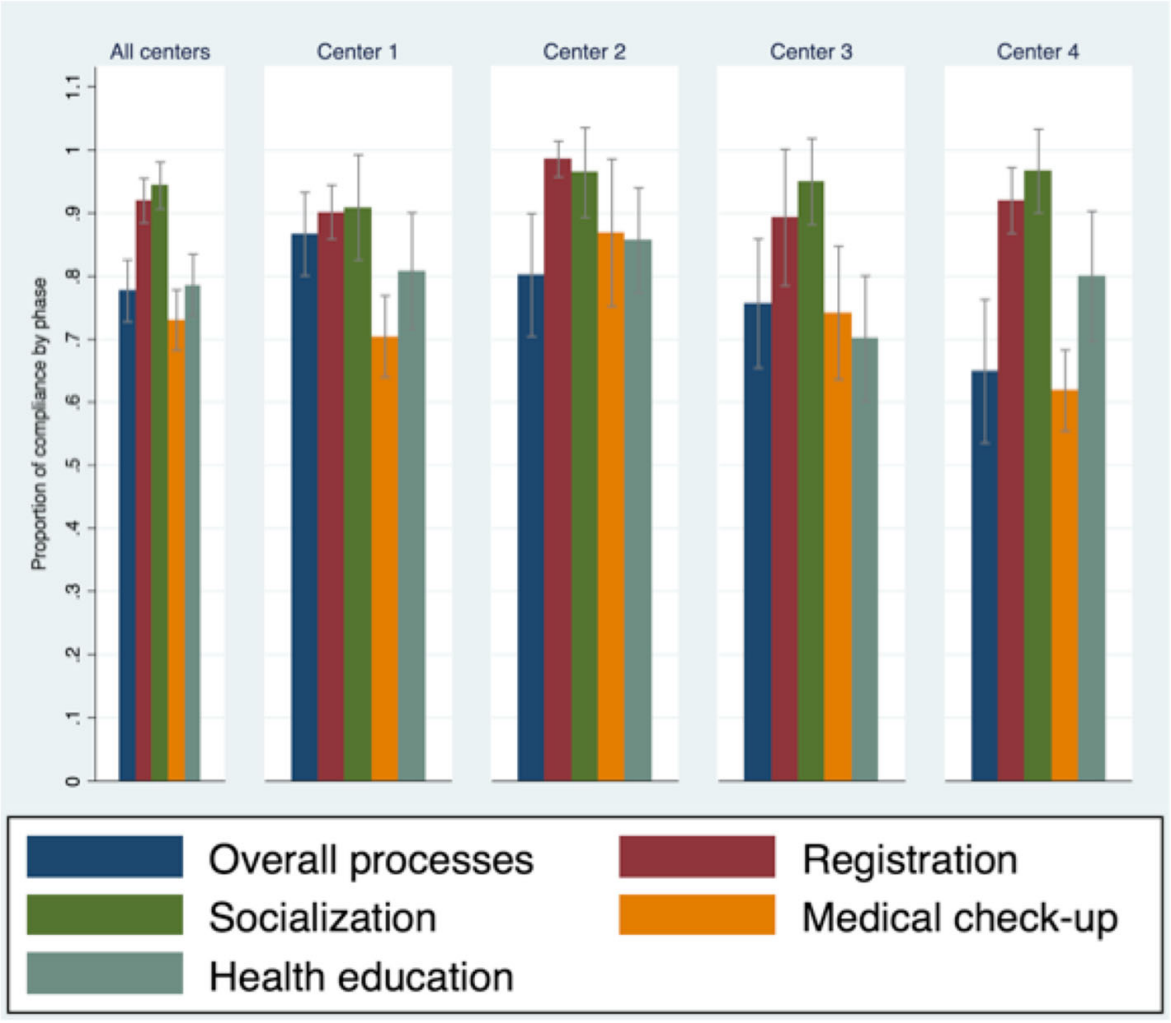
Model fidelity by center. Fidelity estimates. 95% confidence intervals by center

Overall process fidelity for the whole intervention was 83.2% (CI 81.0–85.3). Fidelity by phase varied between 73.1 and 94.4% (Fig. 2). In the first phase the fidelity rate for all centers was 77.7% and the center with the lowest fidelity rate was center 4, within this phase, the item inquiring whether the facilitators introduced themselves in a friendly non-hierarchical way had the lowest fidelity rates with even 0% in center 2 and 33.3% in center 3. Another item with low fidelity in center 4 was the one that asks about a stable number of women in all sessions. In phase 2, involving taking and recording vital signs, fidelity reached 92% for all centers and the item with the lowest fidelity was in center 1 and was about the session beginning on time (31.3%). Socialization phase (Phase 3) reached 94.4% fidelity for all centers and was the phase with the highest fidelity. The fourth phase had 73.1% fidelity for all centers and the center with the lowest fidelity was center 4 (61.9%). The item referring to the *use of music to enhance privacy,* had the lowest fidelity across sites (0–71.4%). In phase 5, health education, fidelity for all centers was 78.6% and the item *the facilitator guided but did not controlled the conversation* had the lowest fidelity, with a range from 21.5% in center 3 to 57.1% in center 2 (Fig. 2).

Table 2 (Supplemental material Table S3) presents results by facilitation team. In phase 1 the item focused on whether the *facilitators introduced themselves in a friendly non-hierarchical way* had the lowest fidelity: 0% for team 3. In this same phase, team 6 had very low fidelity rates in the items: *facilitator is not present during the entire session* (25%), *facilitator is the same that initiated the group* (37.5%) and *number of women in each group* which means group stability (25%). For phase 2, the lowest fidelity belongs to team 1, with 11.11% for the item inquiring if the session began on time. Another result to highlight is in phase 4 where processes during the medical check-ups are measured. This phase includes three items: *space is provided for medical check-ups, check-ups are always conducted in the same space* and if *music is used to enhance privacy.* The first item had a 100% fidelity for all teams and the second one ranged from 75 to 100%, however in the third item that refers to the use of music, 4 teams had a fidelity of 0%, another team has 8.33% and the other two teams had fidelity above 70%. In phase 5 the item with the lowest fidelity for all teams was whether *the facilitator guided but did not control the conversation* where teams 4, 5 and 6 had fidelity lower than 25%.

**Fig. 3:**
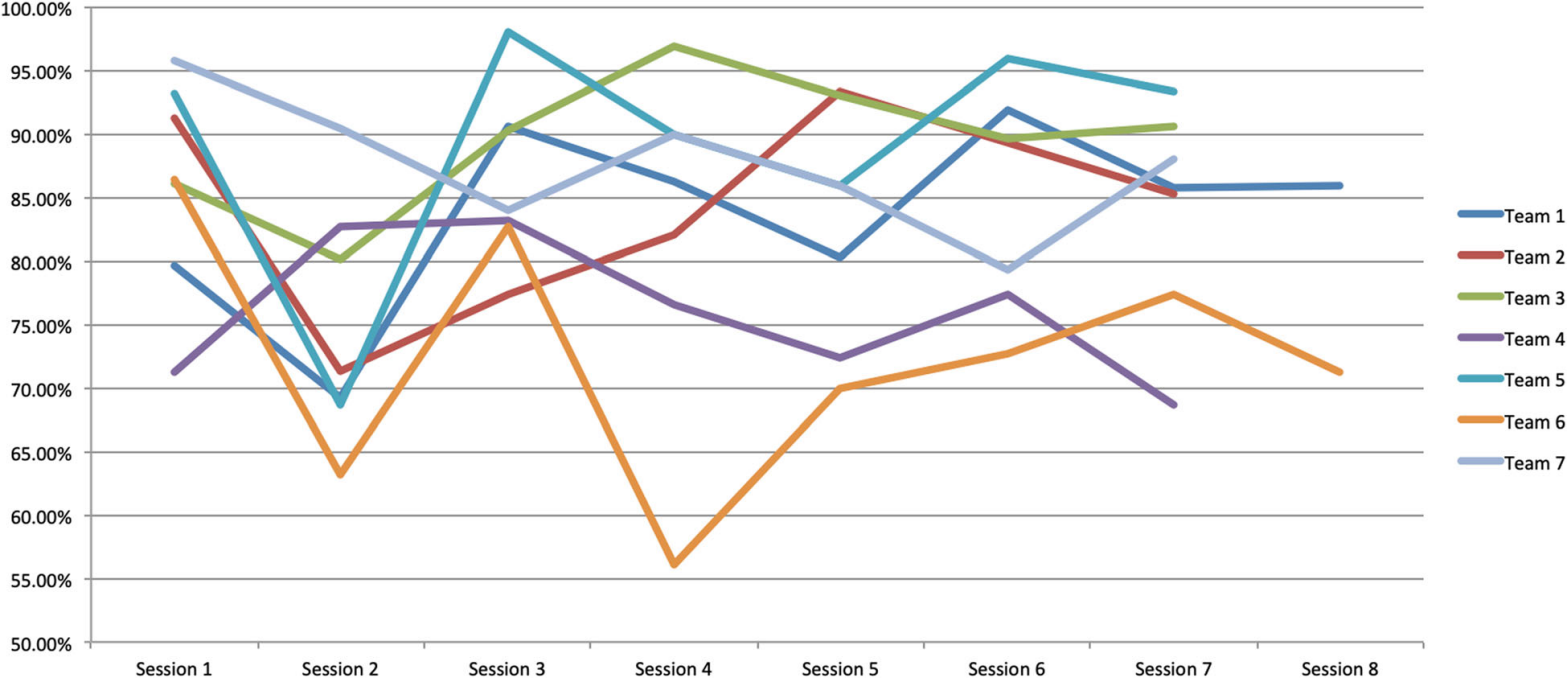
Fidelity per session. Teams 1, 3 and 4 guided two groups each one. Here are presented means between both groups

Figure 3 shows fidelity by session for each one of the seven facilitation teams; we do not observe overall patterns, but most teams experience a reduction in fidelity in session 2, compared with session 1.

## Discussion

We report on process fidelity to the essential elements of CenteringPregnancy during the implementation of an adapted G-ANC model in Mexico. We find that generally the model can be implemented with high fidelity, but that variability exists across elements. For example, items inquiring whether women are taught how to take their own vital signs, space is adequate for taking vital signs, conversational circle is formed and whether space is provided for medical check-ups had the highest overall fidelity (100%) and items inquiring if music is used to enhance privacy and whether facilitator guides but does not control the conversation had the lowest (27.3 and 40% respectively). Fidelity also varied by phase of the intervention and by site. These processes are clearly related to structural and behavioral issues; for example in the structural side, use of music to enhance privacy and beginning the session on time that had low fidelity. For behavioral issues, introducing themselves in a friendly non-hierarchical way and whether the facilitator guided but did not control the conversation had low fidelity.

In comparison to the only literature we found specifically presenting an evaluation of fidelity to the G-ANC model [14], our results demonstrate greater process fidelity. Our overall process fidelity was 83.2%, while Novick et al. reported 77%. However, although data collection was carried out by observation in both cases, different instruments were used. We used one with 28 items, while Novick et al. analyzed only two items: *To what extent was the group session didactic* vs. *facilitative?* And *How much were group members involved and connected?* These two items are similar to the items we included in phase 5, health education, where fidelity was 78.6%, very similar to Novick’s results. Another difference between our studies was the structure of the measurement items. Novick et al. utilized a range of responses for both items, which varied between one and ten points. In our case, we sought absolute or total process fidelity and thus transformed the original Likert-scale items into binary measures.

In our results, the high levels of fidelity in phases 2 and 3, which measure compliance with process during the taking and registering of vital signs and socialization, respectively, suggest that self-care and the building of networks were clearly understood by both the health providers who facilitated sessions and by the expectant mothers. This high level of fidelity during the phases involving the taking and registering of vital signs suggests a certain capacity for change in the role of women towards being more active and involved in their health care. The high degree of compliance with the process of network building indicates awareness on the part of providers of the benefits of socialization among participants.

The processes with low fidelity suggest infrastructure and resource problems more than a lack of adherence to the model in its essential elements. For example, beginning late was partly due to the fact that some centers did not have space within the center and external space was used; this implied transportation time and sometimes waiting until other activities were finished. It is also worth mentioning that even with late start time, group care was likely more punctual than traditional individual care where women experience very long wait times. Another example of low fidelity was the use of music to enhance privacy during the individual clinical checks. Not using music reflects lack of resources in centers more than anything else; facilitators did not have a computer or a music player available in health center. Thus, use of music was up to the initiative of the individual facilitator to bring his or her cell phone and a speaker.

On the other hand, our results in the first phase and phase 5 (fidelity with behavioral aspects throughout the entire session as well as during health education) revealed elements where it was difficult to achieve change in the practices of the providers. The items from both phases with the lowest levels of fidelity were those inquiring whether *the facilitator introduced her/himself in a friendly non-hierarchical way* and *guided but did not control the conversation* - both pointing to the hierarchical medical culture in Mexico [23] and underscoring the verticality in the relationship between doctor and patient. Our results demonstrate the difficulty in changing medical culture [24], and indicate a need for training and approaches more focused on achieving the desired horizontality. Integrating other types of health providers could help with this culture change. The group care model originated with midwives [1], and a horizontal, participatory approach may be more intuitive for midwives than for doctors.

Although the concept of fidelity could be seen as contrary to adaptation, literature suggests that identifying and preserving essential elements of the intervention is compatible with adapting an intervention, [25] and that is what we did in the present study. This approach confirms the importance of evaluating fidelity even in adapted interventions, recognizing that change is a constant as Chambers et al. affirm in their dynamic sustainability framework [26]. According to this framework, authors cite evidence in support of the need to examine the fit between the practice setting and the intervention and make changes necessary to improve the integration of the intervention into ongoing care processes [26]. Authors emphasize that this is consistent with the institutional theory of organizations, which argues that the final stage of innovation requires the “institutionalizing” of the new practice so that it becomes a working part of the organization [26, 27]. Attention to this fit, through ongoing assessment and quality improvement efforts, should improve sustainment and ultimately identify opportunities for intervention improvement [26]. In our study, for example, the presence of music to enhance privacy is one of the components of the intervention that changed and/or was adapted during implementation. Music was included in the checklist, however observers noticed that facilitators will almost never accomplish that due to lack of resources and that privacy remained unchanged, so in the continuous adaptation process music would be a good candidate to be excluded.

One of the limitations of our study was that the number of items per phase was not proportional; however, the averages by phase took this into account. We recognize that there could be some bias due to changes in the behavior of the facilitation team, as a reaction of feeling evaluated. However, the Hawthorne effect was minimized by fostering a rapport between the session facilitators and the observer, generating trust between them, starting with the first facilitator training. Another source of bias could be related to the differences between women in the different health centers; this was a pilot study without sufficient sample to test this hypothesis. However, all 4 sites served low-income women. Another limitation was that the various groups had different observers; nonetheless all pairs of observers were trained in the same way, and the process of completing the instruments was homogeneous across all groups. We analyzed all items as equally important according to the theory of the model that affirm that all essential elements included in the checklist items are equally significant. However, we recognize that not all items of the checklist may be of equal importance to the success of implementing G-ANC model. A strength of our study was that the instrument utilized allowed for evaluating a large number of processes.

## Conclusions

In conclusion, our study shows G-ANC model fidelity to the essential elements of the model. Further, we identified elements that require special attention - processes where it was most challenging to achieve fidelity during the implementation of the G-ANC model. Our results show that infrastructure (beginning on time; use of music) and behavioral (guided but did not control the conversation*)* aspects are the hardest to achieve high fidelity.

This study serves as an example of the trade-off between adapting a complex intervention and fidelity to the model. Our results about fidelity will be useful when refining, replicating, and scaling this intervention.

## Supplementary information

**Additional file 1: Table S1.** Essential Elements of the Centering Healthcare Model of Group Care and phases in which they occur. **Table S2.** Implementation of the G-ANC model in Mexico. **Table S3.** Process fidelity during the implementation of Group Antenatal Care in Mexico: results by phase and for the entire intervention according to trained facilitation teams.

## Data Availability

The datasets during and/or analysed during the current study available from the corresponding author on reasonable request.

## Abbreviations

CP: CenteringPregnancy
G-ANC: Group Antenatal Care
