# Supplementary material for: Evaluating process fidelity during the implementation of Group Antenatal Care in Mexico
Source: BMC Health Serv Res. 2020 Jun 18;20:559. doi: 10.1186/s12913-020-05430-z (PMC7301482; doi:10.1186/s12913-020-05430-z)
Supplement: Supplementary file 1 — Additional file 1: Table S1. Essential Elements of the Centering Healthcare Model of Group Care and phases in which they occur. Table S2. Implementation of the G-ANC model in Mexico. Table S3. Process fidelity during the implementation of Group Antenatal Care in Mexico: results by phase and for the entire intervention according to trained facilitation teams. [file 12913_2020_5430_MOESM1_ESM.docx]

**Supplemental material**

| **Table S1. Essential Elements of the Centering Healthcare Model of Group Care and phases in which they occur** | | | |
| --- | --- | --- | --- |
| Essential Element | Process | Phases | Maintained or adapted |
| 1. Group members, including facilitators and support people, are consistent | - New women may join an existing group with the group’s agreement. - Facilitators are present for the entire session. - There is an emergency backup plan for facilitators. - Professional students are supervised and consistent throughout all sessions. - Children are not present during circle-up (other than CenteringParenting babies). | Phase 1 Cross-cutting processes | Adapted: Sometimes, because of administrative or scheduling problems the facilitating team could not assist to the session and if the center had more than one facilitating team the other one had to intervene |
| 2. Group size is optimal to promote the process | - CenteringPregnancy groups are between 8-12 women. - CenteringParenting groups are between 6-8 mother-baby pairs. - There is an appropriate balance of women and providers. | Phase 1 Cross-cutting processes | Adapted: Due to recruitment problems sometimes we had more than 12 members in a cohort |
| 3. Women are involved in self-care activities | - Women collect and record their own health information | Phase 2 Registration and taking of vital signs | Maintained |
| 4. Opportunity for socializing within the group is provided | - There is some downtime when women can socialize | Phase 3 Socialization | Maintained |
| 5. Health assessments (check-ups) are conducted within the group space | - The assessment area is set up to ensure privacy (position and level of exam area, music, plants, other soft barriers) | Phase 4 Medical check-ups | Adapted: Because of cultural aspects, we adopted a screen to divide the circle and the assessment, and use a little cot instead of the mat. |
| 6. A facilitative leadership style is used | - Facilitators guide but do not control the discussion and refer questions to the group. - Women voluntarily share information, ideas, personal feelings or experiences. - Group guidelines are established. - Facilitators dress casually. - Women sign a confidentiality agreement. | Phase 5 Health education | Maintained |
| 7. Each session has an over-all plan, but emphasis will vary | - Centering materials are used to plan and evaluate sessions. | Phase 5 Health education | Adapted: We created a curriculum that reflected content mandated by the Ministry of Health. |
| 8. The group is conducted in a circle. | - There are no observers outside the circle. - Centering space is private and conducive to group sharing. - Seating is around an open space. - The circle up of the group does not happen until all facilitators, women and support people are in the circle | Phase 5 Health education | Maintained |
| 9. There is on-going evaluation of outcomes | - Outcomes, model fidelity, and sustainability are reported in CenteringCounts. (not necessary for Mexico) - Facilitators regularly assess the group experience. | Outside any phase | Maintained |
| *Adapted from Essential Elements of CenteringPregnancy (7) | | |  |

**Table S2. Implementation of the G-ANC model in Mexico**

| **Implementation of the model in Mexico** | **Description** |
| --- | --- |
| Steps of the feasibility study | Period: from June 2016 to august 2018.   1. **Ministry of health buy-in and training of Mexican team.** We negotiated with stakeholders and decision makers of the 2 state ministries of health (Morelos e Hidalgo). At the same time, 2 members of our research team attended to United States, for a 2-day intensive training workshop in CP to become our national experts. We also began our collaboration with a CP expert in United States. 2. **Adaptation of content and format of the CP model**. We created a curriculum that reflected content mandated by the Ministry of Health. We also adapted the originals CP materials. 3. **Site selection process**. We realized diagnostic studies, summarizing the physical space, patient volume and personnel on site. With these studies, the help of our CP expert and the guide of the Ministry of Health we selected the health centers where we implemented the model. Besides all these criteria we consider the willingness of center staff to participate. 4. **Initial training of health center staff.** We trained health personnel (physicians, nurses and social workers) of the chosen sites. This training also worked as a first training of trainers to our national experts. 5. **Pilot.** We began with the implementation in 2 sites, one in each state. We delivered the materials and follow and feedback the recruitment process. Research team was involved accompanying the development of both groups. 6. **Implementation**: We took advantage of all our acquired knowledge from the previous stages to extend the implementation to all chosen centers. |
| Number of centers by state | Morelos: 3 health centers of the first jurisdiction of the Ministry of Health in the state.  Hidalgo: 1 health center that belong to the third jurisdiction of the Ministry of Health in the state.  *All centers belong to the Ministry of Health that provides health care to the population that does not have any other social security provider. |
| Health personnel by site | During the whole study 23 health providers were trained (3 workshops of 2 days each). 15 of the trained providers became facilitators of groups:   \| Site/center \| State \| # Facilitators \| Profile \| \| --- \| --- \| --- \| --- \| \| Center 1 \| Morelos \| 3 \| 2 physicians and 1 nurse \| \| Center 2 \| Morelos \| 2 \| 1 physician and 1 nurse \| \| Center 3 \| Hidalgo \| 5 \| 2 physicians, 2 nurses and 1 social worker \| \| Center 4 \| Morelos \| 5 \| 2 physicians, 2 nurses and 1 health promoter (psychologist) \| |

|  | **Table S3. Process fidelity during the implementation of Group Antenatal Care in Mexico: results by phase and for the entire intervention according to trained facilitation teams** | | | | | | | | | | | |  |
| --- | --- | --- | --- | --- | --- | --- | --- | --- | --- | --- | --- | --- | --- |
| Phases | | All sessions | Facilitation teams | | | | | | | | | |  |
|  |  |  | Center 1 | | Center 2 | | Center 3 | | | Center 4 | | |  |
|  |  |  | Team 1 | Team 2 | | Team 3 | | Team 4 | Team 5 | | Team 6 | Team 7 |  |
|  |  | N=71 | N= 15 | N= 7 | | N= 14 | | N= 13 | N= 7 | | N= 8 | N= 7 |  |
| Overall process fidelity | | 83.18 | 83.19 | 83.52 | | 88.17 | | 75.00 | 82.61 | | 69.19 | 88.76 |  |
| Phase 1 Cross-cutting processes | | 77.71 | 83.56 | 95.24 | | 80.56 | | 69.70 | 83.72 | | 52.00 | 80.95 |  |
| Names of facilitators are visible | | 80.00 | 100.00 | 100.00 | | 90.00 | | 55.56 | 71.43 | | 62.50 | 83.33 |  |
| Names of participating women are visible | | 92.86 | 100.00 | 100.00 | | 100.00 | | 75.00 | 85.71 | | 87.50 | 100.00 |  |
| Cycle is always developed in the same place | | 86.79 | 87.50 | 100.00 | | 100.00 | | 75.00 | 100.00 | | 62.50 | 83.33 |  |
| Facilitator shows credentials (sessions 1 and 2 only) | | 50.00 | 50.00 | 100.00 | | 0.00 | | 20.00 | 100.00 | | 100.00 | 100.00 |  |
| Facilitator is present during the entire session | | 68.57 | 80.00 | 71.43 | | 61.54 | | 76.92 | 71.43 | | 25.00 | 85.71 |  |
| Facilitator is the same that initiated the group | | 84.06 | 85.71 | 100.00 | | 100.00 | | 84.62 | 85.71 | | 37.50 | 85.71 |  |
| Number of women who do not show significant changes | | 66.15 | 71.43 | 100.00 | | 69.23 | | 70.00 | 85.71 | | 25.00 | 42.86 |  |
| Phase 2 Registration and taking of vital signs | | 91.97 | 87.50 | 91.67 | | 98.41 | | 88.89 | 97.22 | | 92.68 | 91.43 |  |
| Session begins on time | | 68.42 | 11.11 | 57.14 | | 90.00 | | 77.78 | 100.00 | | 87.50 | 57.14 |  |
| Women are taught how to take their own vital signs (session 1) | | 100.00 | 100.00 | 100.00 | | 100.00 | | 100.00 | 100.00 | | 100.00 | 100.00 |  |
| Women weigh themselves | | 97.14 | 100.00 | 100.00 | | 100.00 | | 91.67 | 85.71 | | 100.00 | 100.00 |  |
| Women check their own blood pressure | | 98.57 | 100.00 | 100.00 | | 100.00 | | 91.67 | 100.00 | | 100.00 | 100.00 |  |
| Women record their own gestational information | | 94.20 | 100.00 | 100.00 | | 100.00 | | 81.82 | 100.00 | | 75.00 | 100.00 |  |
| Space is adequate for taking vital signs | | 100.00 | 100.00 | 100.00 | | 100.00 | | 100.00 | 100.00 | | 100.00 | 100.00 |  |
| Phase 3 Socialization | | 94.37 | 90.00 | 92.86 | | 96.43 | | 92.31 | 100.00 | | 93.75 | 100.00 |  |
| Conversational circle is formed | | 100.00 | 100.00 | 100.00 | | 100.00 | | 100.00 | 100.00 | | 100.00 | 100.00 |  |
| Opportunity for socializing is provided | | 88.73 | 80.00 | 85.71 | | 92.86 | | 84.62 | 100.00 | | 87.50 | 100.00 |  |
| Phase 4 Medical check-up | | 73.09 | 68.57 | 66.67 | | 89.47 | | 58.82 | 100.00 | | 58.33 | 66.67 |  |
| Space is provided for medical check-ups | | 100.00 | 100.00 | 100.00 | | 100.00 | | 100.00 | 100.00 | | 100.00 | 100.00 |  |
| Check-ups are always conducted in the same space | | 94.20 | 100.00 | 100.00 | | 100.00 | | 84.62 | 100.00 | | 75.00 | 100.00 |  |
| Music is used to enhance privacy | | 27.27 | 8.33 | 0.00 | | 71.43 | | 0.00 | 100.00 | | 0.00 | 0.00 |  |
| Phase 5 Health education | | 78.61 | 83.22 | 75.36 | | 85.40 | | 72.66 | 65.71 | | 66.25 | 95.71 |  |
| Facilitator guides but does not control the conversation | | | 40.00 | 46.67 | 42.86 | | 57.14 | | 25.00 | 14.29 | | 0.00 | 85.71 |
| Facilitator addresses group eliciting responses | | | 69.01 | 66.67 | 57.14 | | 85.71 | | 61.54 | 42.86 | | 62.50 | 100.00 |
| Group feels more like a peer group than a class | | | 80.28 | 80.00 | 71.43 | | 92.86 | | 76.92 | 42.86 | | 87.50 | 100.00 |
| Women talk during most of the session | | | 81.42 | 100.00 | 57.14 | | 100.00 | | 58.33 | 57.14 | | 75.00 | 100.00 |
| Facilitator sits within the circle and remains seated | | | 84.51 | 93.33 | 100.00 | | 71.43 | | 92.31 | 100.00 | | 50.00 | 85.71 |
| Facilitator has prepared the theme to be developed during the session | | | 88.57 | 92.86 | 85.71 | | 100.00 | | 84.62 | 100.00 | | 50.00 | 100.00 |
| Facilitator observes a methodology for developing the theme | | | 98.55 | 100.00 | 100.00 | | 100.00 | | 92.31 | 100.00 | | 100.00 | 100.00 |
| Facilitator values the contributions of every group member | | | 81.69 | 80.00 | 71.43 | | 92.86 | | 76.92 | 71.43 | | 75.00 | 100.00 |
| Information, ideas, feelings and experiences are shared | | | 75.71 | 93.33 | 71.43 | | 76.92 | | 53.85 | 57.14 | | 87.50 | 85.71 |
| Participants understand the theme addressed | | | 85.71 | 80.00 | 100.00 | | 76.92 | | 100.00 | 71.43 | | 75.00 | 100.00 |
| N = Number of sessions | | |  |  |  | |  | |  |  | |  |  |
